# Biological evidences for successive oogenesis and egg-laying of *Matsumurasca onukii*

**DOI:** 10.1371/journal.pone.0263933

**Published:** 2022-02-17

**Authors:** Yali Chang, Yuxian Xing, Yanan Dong, Xiwang Li, Songbo Lin, Yi Chen, Xiaoling Sun

**Affiliations:** 1 Tea Science Department, Henan Engineering Research Center of Tea Processing and Testing, Henan Key Laboratory of Tea-plants Comprehensive Utilization in Southern Henan Province, Xinyang Agriculture and Forestry University, Xinyang, Henan, China; 2 Key Laboratory of Tea Biology and Resources Utilization, Ministry of Agriculture, Tea Research Institute, Chinese Academy of Agricultural Sciences, Hangzhou, Zhejiang, China; Zhejiang University, CHINA

## Abstract

Tea plant (*Camellia sinensis*) is one of the most important horticultural cash crops, and tea green leafhopper (*Matsumurasca onukii*) is an extremely harmful sap-sucking pest of tea plant. Serious generation overlapping, which is mainly caused by the long oviposition period, leads to poor control effect of pesticides on this pest in the tea plantation. But the intuitive evidences of continuous oogenesis and egg-laying of this pest are still lacking, which seriously hindered the development of genetic control methods. Here, we clarified the main structures of the inner reproductive system of tea green leafhopper female adult. Oviposition behaviors were monitored as well, and six oviposition steps were recorded. According to the maturity of oocytes, the maturity stages of the reproductive system under different copulation periods were classified into 4 stages. For female adults at stage IV, mature and immature oocytes were presented simultaneously, and the developmental levels of oocytes were asynchronous among different ovarioles. The proportion of gravid females with mature oocytes significantly increased when the continuous copulation time was prolonged. In sync with the development of the ovary maturity, female adults started to slightly deposit eggs at the 5th day, and then increased dramatically. In addition, we found that, whether mature or immature, oocytes in the ovarioles always emitted green fluorescence under blue light excitation, which in turn provide solid proof for the new egg detection method from the insect physiology point of view.

## Introduction

Oogenesis and oviposition have been considered to be the key physiological phases of oviparous insect females in their reproductive process [1]. Investigating the biological characteristics, revealing the underlying mechanisms of oogenesis and oviposition may help to develop new integrated pest management approaches. Recently, RNA interference (RNAi)-mediated knockdown of certain genes has been verified to influence the development of ovary and oocyte maturation [2, 3]. For example, after knockdown of *Krüppel homologue 1*(*kr-h1*), the development of oocyte in *Locusta migratoria*, *Bactrocera dorsalis*, and *Chilo suppressalis* was significantly suppressed [4–6].

The internal reproductive systems of female herbivores have been known as the site of oocyte development and zygote formation [7]. Herbivore oocytes developed and matured in the ovarioles after females developed into a certain stage, formed the zygotes after receiving the sperm from spermatheca, then the zygotes entered the vagina or the reproductive cavity, and the mature eggs were laid outside finally [8]. It is worth noting that the biological characteristics of oogenesis and egg-laying mode are specific with different herbivorous insect species. For example, in rice brown planthopper (*Nilaparvata lugens*, BPH), several milky banana-shaped mature oocytes appeared in the terminal follicles of ovarioles when the female ovary was at stage III, and then several fully mature oocytes appeared successively in the female adult abdomen [9, 10]. BPH usually laid separate egg masses along the same leaf vein and each mass had 4.7 eggs in average [11]. The rapid and sequential development characteristics of oocytes were responsible for its robust fecundity, which was supposed to be the reason of frequent outbreaks of BPH in the field [9, 10, 12]. However, the situation is completely different in the glassy-winged sharpshooter (*Homalodisca coagulata*, GWSS). When the maturity status of GWSS ovary was at the rank of 3, 4, or 5, each ovariole owned a single sausage-shaped mature oocyte or two immature oocytes simultaneously, and the maturity levels of oocytes in each ovariole were almost synchronous [13]. Since there were 8–10 ovarioles in a single ovary of GWSS, gravid female nurtured around 20 mature oocytes concurrently [14, 15]. Usually, GWSS female laid eggs side-by-side in batches of 10–15 forming a “blister” under the epidermis of the leaf [14, 16]. Therefore, it is necessary to study the morphological structure of the reproductive system of female insect adult, and then explore the oogenesis and egg-laying pattern of a certain species, which will help for improving the strategies of prediction and forecast. As we all know, insect oviposition is another important aspect of reproduction, which greatly affects its population level in an ecosystem [1, 17]. Normally, the oviposition behavior was directly observed and recorded by video [15, 18–20]. The egg density and location was investigated directly under the stereomicroscopy or observed by naked eye. Obviously, combined using of the above methods would be helpful to reveal the biological characteristics of oviposition more comprehensively for a certain herbivorous species, especially for the small insect with concealed oviposition habit.

Tea plant (*Camellia sinensis*) is one of the most important horticultural cash crops in almost 30 countries. Tea green leafhopper (*Matsumurasca onukii* (Mstauda, 1952)) is an extremely harmful sap-sucking pest of tea plant, usually has ten generations per year and overwinters as an adult [21–23] (S1 Fig). The generations of *M*. *onukii* in tea plantation are extremely overlapped, which mainly attribute to the long oviposition period, thus the control effect of chemicals on *M*. *onukii* is not as effective as expected [21]. The outbreaks of *M*. *onukii* can greatly reduce tea yield and quality in China, Japan and Viet Nam, especially in China [21, 23]. In the lifespan of an adult female of *M*. *onukii*, more than 40 eggs were deposited for up to 20 d; the female adults lay eggs singly between cortex and xylem in the tender part of the tea plant, either in the stems or in the main vein of leaves [21, 23]. Although several traits of *M*. *onukii* egg-laying, including egg distribution, oviposition circadian rhythm, and ecological influence factors of oviposition preference, have been reported primarily, the intuitive evidences of continuous oogenesis and egg-laying are still lacking [23–25]. In the present study, based on the physiological structure of the inner reproductive system of *M*. *onukii*, the ovary maturity of female adults under different copulation time and the egg number corresponding to the same mating status were clarified and compared firstly; secondly, the oviposition behavior was monitored using a self-designed video monitor; thirdly, daily and circadian rhythms of oviposition were reported as well. Our research will greatly enrich the understanding the biological characteristics associated with oviposition of tea green leafhoppers.

## Materials and methods

### Insects

*M*. *onukii* adults were collected from the tea plantation of Tea Research Institute, Chinese Academy of Agricultural Sciences, Hangzhou, Zhejiang province, China (30°10’N, 120°56’E). Insect adults were kept into a cage (nylon, 75×75×75 cm, S2 Fig), which was placed in a walk-in climate chamber (25 ± 2°C,70 ± 5% relative humidity, 14:10 light/dark), and reared with timely replaced fresh tender tea shoots. After rearing for two generations, the 5th instar nymphs were carefully collected from cages and raised with a tender stem in a 10 mL plastic centrifuge tube individually (S2 Fig).

### Treatments

The tube-rearing nymphs were checked at 9:00 am every day, and then new eclosion insect were regarded as 1-d-old adult. The genders were identified according to the morphological characteristics of external genitalia [26]. Then, four male and four female adults were carefully introduced into a self-designed glass tube to perform the continuous copulation (S2 Fig). One fresh tea shoot was inserted into the flower mud from the top hole, replaced every day. Females *M*. *onukii* were collected at 9:00 am at 1, 3, 5, 7 and 9 d after the start of the mating separately, of which the inner reproductive systems were dissected and observed under the stereomicroscope (SZ61, Shanghai BAGUAN Instrument Technology Co., Ltd., China), then photographed with the software BasedCam 2.0. At the same time, the egg number in the tea shoots corresponding to the same mating status was checked using the blue light detection method (BLDM) [23, 27]. Each time point was replicated 19–28 times.

### Oviposition behavior

**Fig 1:**
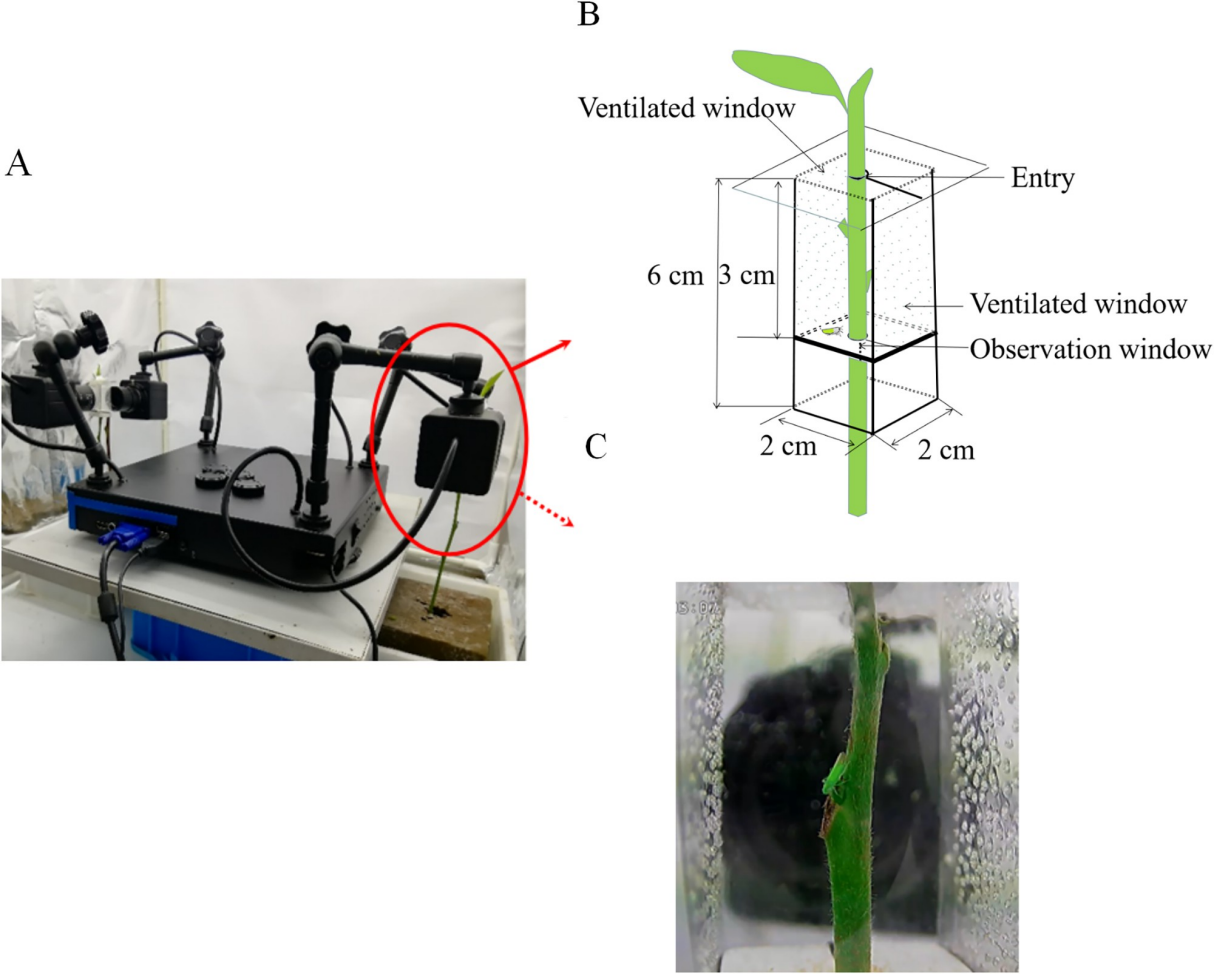
Device for monitoring oviposition behavior. (A) Panoramic view of the recording device; (B) Diagram of container; (C) Inside real view of the container.

The oviposition behavior of tea leafhopper female was investigated using a self-designed insect behavior video monitor (Fig 1). During this study, a single *M*. *onukii* gravid female (with 8 d successive copulation experience) was placed into a transparent observation device (high transparency PET mylar, 2×2×6 cm, Fig 1B and 1C), supplemented with a tender tea shoot without leaves. A total of 10 *M*. *onukii* gravid females were monitored individually.

### Oviposition rhythm

The oviposition daily rhythm and circadian rhythm were investigated in the glass tubes (S2 Fig). For daily rhythm investigation, four female and four male adults at 1 d age were confined in the same glass tube, supplied with a tender tea shoot; the tea shoot was checked at 9:00 am every day. Four gravid female adults with 8 d copulation experience were used for circadian rhythm investigation, as we found that the egg amount laid by the female adult at the 8th day is relatively high in the daily rhythm investigation. Eggs in a tea shoot were checked every 6 h from 6:00 am to 24:00 pm by using BLDM. 20 biological repetitions were conducted.

### Data analysis

All statistical analyses were performed by using the Statistical Package for the Social Science (SPSS 20.0). The ratios of tea green leafhopper female adults at different developmental stages, numbers of eggs laid by female adults with different copulation time were analyzed via one-way ANOVA. If the ANOVA analysis showed significant (*P* < 0.05), Tukey’s honest significant difference (HSD) post-hoc test was used to detect difference between groups. Student’s *t*-test was used for comparing difference among different treatments.

## Results

### Morphological structure of the inner reproductive system of female adult

**Fig 2:**
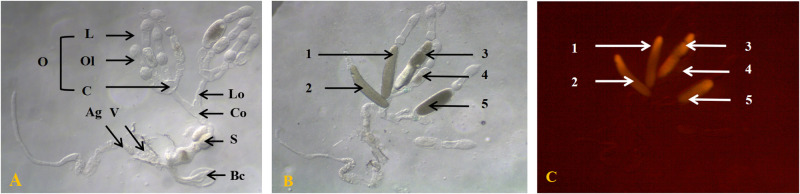
The inner reproductive system of *Matsumurasca onukii* female adult under stereomicroscopy with cold light (A, B) and with blue light (C). L, terminal filament; C, ovarian calyx; Ol, ovariole; O, ovary; S, spermatheca; Ag, accessory gland; Lo, lateral oviduct; Co, common oviduct; Bc, bursa copulatrix; V, vagina; 1 and 2 were mature oocytes; 3, 4, and 5 were immature oocytes.

The basic morphological structures of the inner reproductive system of tea green leafhopper female adult were preliminarily presented through hand-painting picture [28]. With the development of technology, it is coming true to present the inner reproductive system clearly and precisely. Here, we took pictures of the inner reproductive system of tea green leafhopper female under the stereomicroscopy with cold light and blue light (Fig 2A and 2C). The main structures of inner reproductive system of tea green leafhopper female were composed of a pair of ovaries, a pair of lateral oviducts, a median oviduct, a spermatheca, a bursa copulatrix, and a single accessory gland, which were mainly named after related studies [28–30]. Among them, two ovaries were symmetrical, and one ovary was composed of four telotrophic ovarioles. Each ovariole owned a single gracile terminal filament at the top, and four terminal filaments converged into a common sling. Each ovariole was connected by an ovarian pedicel to ovarian calyx, which was formed by anterior enlargement of lateral oviduct. The distal ends of two lateral oviducts collectively intersected with the top of median oviduct, forming a ‘Y’ shape. The central enlargement of median oviduct formed the nearly heart-shape spermatheca, of which one side was with two protuberant structures, other side was relatively flat. The relatively thick end of median oviduct was connected with the bursa copulatrix and vagina. The accessory gland was a tubular structure with uneven thickness (Fig 2A).

When the inner reproductive system was in high maturity, mature and immature oocytes were presented simultaneously in the ovary (Fig 2A and 2B), and the results were verified by the BLDM (Fig 2C), which in turn provide solid proof for the egg detection method from the insect physiology point of view [23, 27].

### Morphological changes of the inner reproductive system during oogenesis

**Fig 3:**
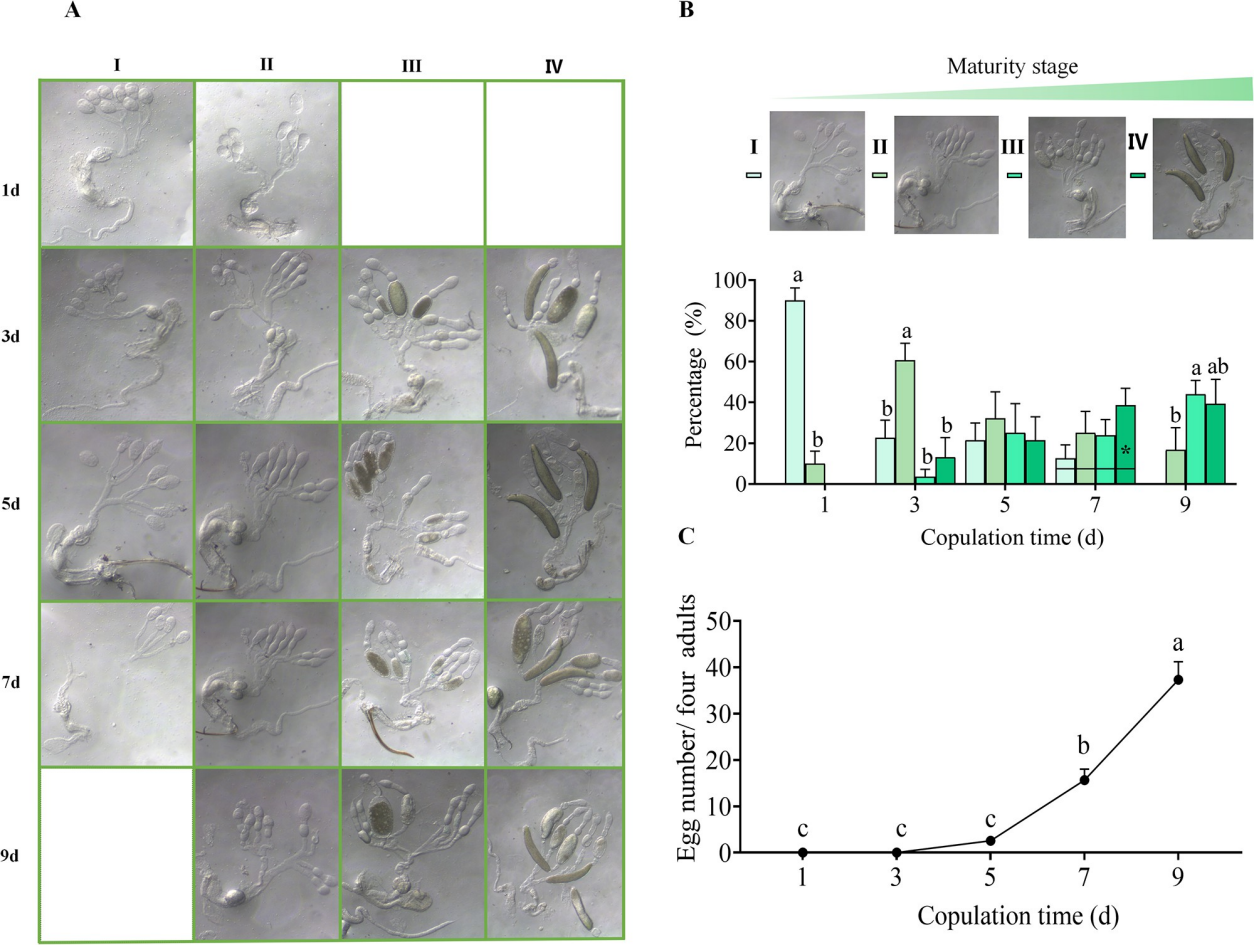
Morphological changes of the inner reproductive system (A), proportion of each maturity stage in different copulation time (B), and egg amounts deposited in the corresponding treatment (C). Stage I-IV referred to the ovary maturity stage I-IV. 1d, 3d, 5d, 7d, and 9d referred to the continuous copulation time. Data are from mean (+SE). For each time point, different letters indicate significant differences among treatments (*P* < 0.05, Tukey’s honest significant difference (HSD) post-hoc test, n = 19–28).

The developmental process for the female inner reproductive system of tea green leafhopper was classified into 5 stages according to the maturity of oocyte [28]. The staged IV, which was defined as each ovariole owning one mature oocyte and one developing immature oocyte simultaneously [28], was not observed in our study. Therefore, the maturity stages of the reproductive systems were slightly modified based on our anatomical results (Fig 3A). For stage I, there is an enlarged transparent ooecium at the top of ovariole, and spermatheca is obviously visible; for stage II, there are two enlarged transparent egg chambers for each ovariole, and a spermatheca is distinctly visible; for stage III, there is normally one immature creamy yellow oocyte in one of the ovarioles, of which yolk started to accumulate, and spermatheca is precisely visible; for stage IV, there are several mature oocytes and immature oocytes in the ovary, and spermatheca is not obvious any more; for stage V, all ovarioles become slender and atrophic, there may be still a single mature oocyte inside.

For 1 d copulation, only 10.0% of females have already finished sexual maturation and the maturity of ovaries was at stage II (*F*_3,16_ = 101.333, *P* < 0.001) (Fig 3B). For 3 d continuous copulation, the ovary maturity of 13.1% of females was at stage IV and several mature oocytes appeared in the ovarioles (*F*_3,24_ = 10.045, *P* < 0.001) (Fig 3B). For 5 d continuous copulation, the proportion of the ovary maturity in each stage was almost even (*F*_3,24_ = 0.175, *P* > 0.05) (Fig 3B). For 7 d continuous copulation, 62.6% of the ovary maturity were at stage III and IV, it reached to 83.3% for 9 d continuous copulation and none of ovary maturity was at stage I (*F*_3,28_ = 1.610, *P* > 0.05, 7d; *F*_3,24_ = 5.415, *P* < 0.05, 9d) (Fig 3B). The proportions of tea green leafhopper females owning mature oocytes reached to 39.29% after continuous 9 d’s copulation (Fig 3B). In sync with the development of the ovary maturity, female adults deposited a few eggs at the 5th day, and then the egg amount increased significantly (*F*_4,95_ = 58.387, *P* < 0.001) (Fig 3C).

Anatomical results exhibited, the asynchronous development of oocytes among different ovarioles became conspicuous when the inner reproductive systems were at stages III, IV (Fig 3A). The maturity of oocytes were different and one of them began to have yolk deposition ahead of others when the inner reproductive system was at stage III. Futhermore, 4–5 fully mature and immature oocytes were shown simultaneously in the ovary when the inner reproductive system was at stage IV (Fig 3A), which could explain the oviposition characteristics of tea green leafhopper to some extent, including daily amount of egg-laying, successive pregnancy, and egg-laying.

### Characterization of oviposition behavior

**Fig 4:**
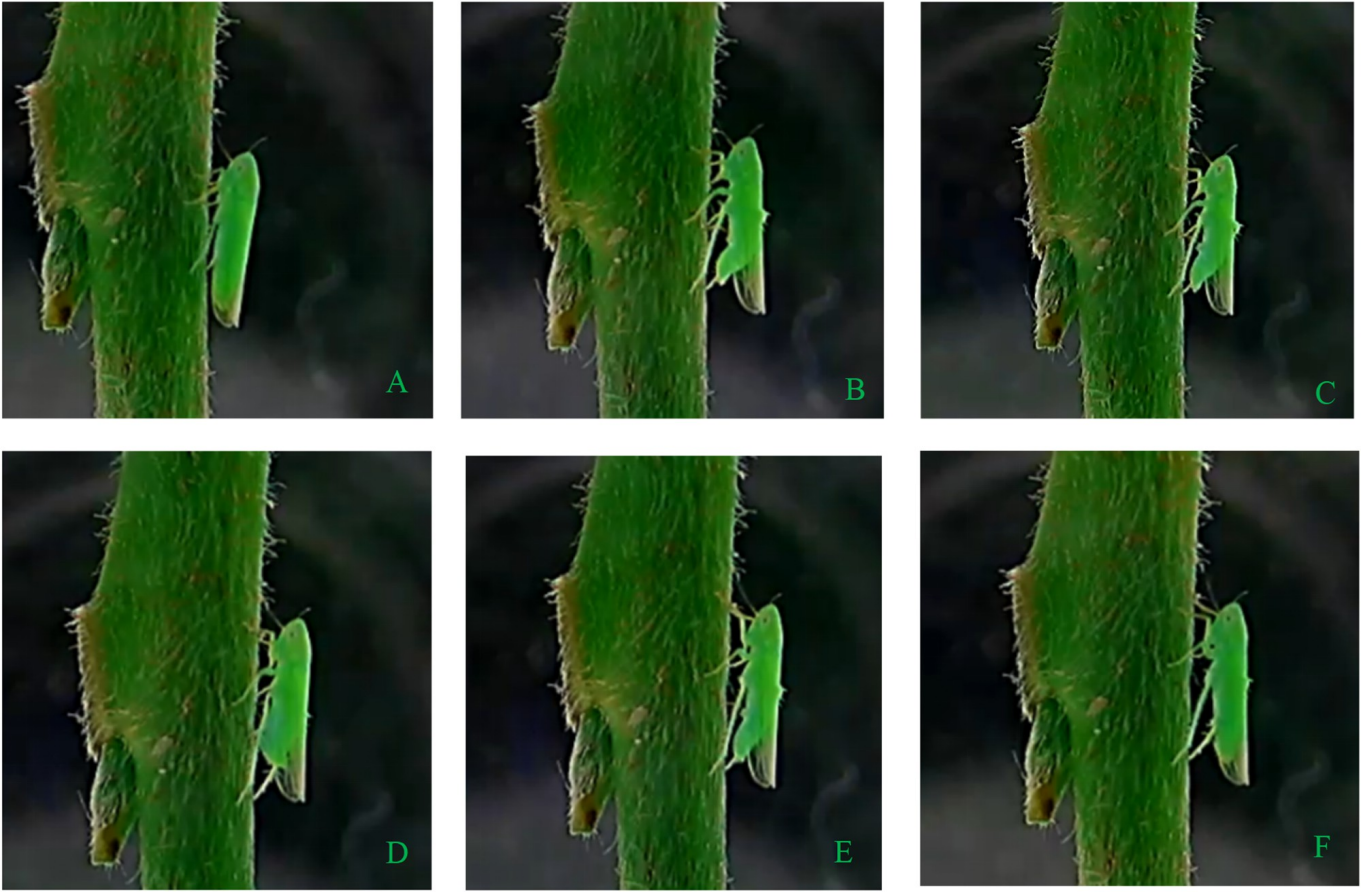
Oviposition behavior of the *Matsumurasca onukii* female adult. (A) Stopping feeding; (B) Abdomen bending and ovipositor extending; (C) Adjusting body gesture and penetrating epidermis; (D) Releasing an egg; (E) Ovipositor withdrawal; (F) Resting.

The oviposition behavior was monitored continuously by self-designed monitor device (Fig 1). Except for the behaviors of body cleaning, crawling, flapping wings, flying, honeydew excretion and egg-laying, *M*. *onukii* female adult almost fed continuously on the tender stem at other times. According to the monitor-record, the whole oviposition process of *M*. *onukii* could be divided into six steps clearly: Step 1) female adult draws out the mouth needle sheath from the tea shoot and stops feeding (Fig 4A); Step 2) moves forward a little bit, bents the abdomen, releases the ovipositor from the sheath, moves forward and afterward gently to form an angle of 90° between ovipositor and tea shoot, then adjusts the body position slightly (Fig 4B); Step 3), ovipositor punctures the epidermal and parenchymal cells with the help of the body shaking, then pushes ovipositor into tea shoot by rhythmically twitching its abdomen and continually adjusting gesture (Fig 4C); Step 4), slightly and continuously shakes the body to assist egg laying (Fig 4D); Step 5), withdraws and ensheathes the ovipositor (Fig 4E); Step 6) after resting for 1–3 min, begins feeding (Fig 4F). The total time from step 2 to 5 lasted for approximately 2 min. More details were exhibited in Fig 4 and S1 Video.

### Dynamic rhythm of oviposition

**Fig 5:**
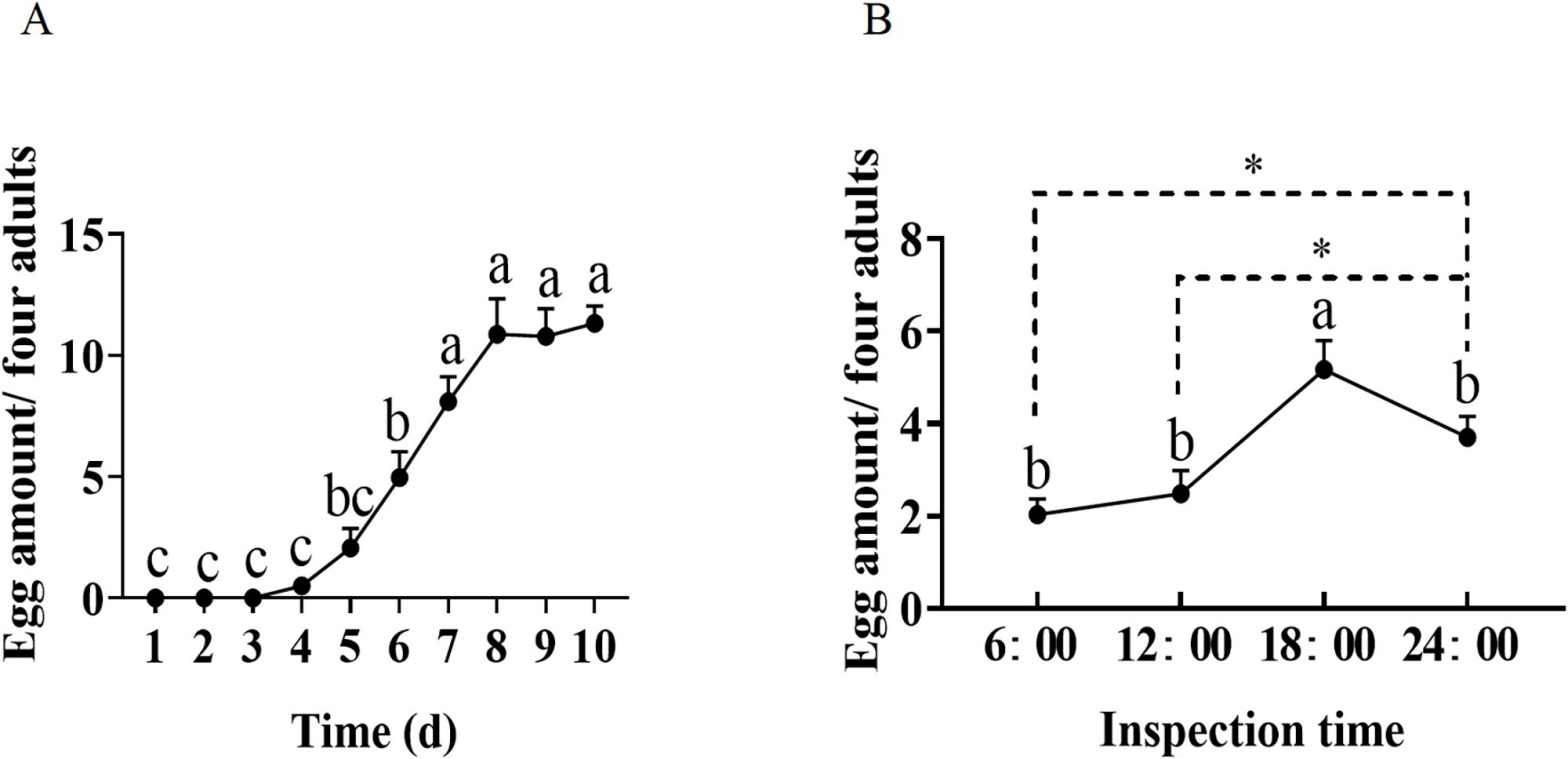
Investigation of oviposition dynamics of daily rhythm (A) and circadian rhythm (B). Data are from mean (+SE). For each time point, different letters indicate significant differences among treatments (*P* < 0.05, Tukey’s honest significant difference (HSD) post-hoc test, *n* = 20). * indicates significant difference at *P* < 0.05 level by student’s *t* test.

In this part, the number of eggs laid by the tea green leafhopper female adults in a self-designed glass tube was inspected every day. In our inspected period, tea green leafhopper females began to lay eggs from the 4th day and reached the oviposition peak at the 7th day, and then lasted to the 10th day (*F*_9,190_ = 35.781, *P* < 0.001) (Fig 5A). Furthermore, there were about 11 eggs laid by four females at the 8th day. Moreover, we found that the tea green leafhopper female adult laid eggs at any time throughout the day, which was observed both under the monitor and the rhythm investigation (Fig 5B). The amount of eggs laid by *M*. *onukii* females at 12:00–18:00 and 18:00–24:00 were significantly higher than those at the other two time periods (*F*_1,38_ = 17.923, *P* < 0.001, Fig 5B), suggesting that tea green leafhopper may prefer to lay eggs during this period.

## Discussion

Cicadellidae is a very large family. More investigations revealed that the number of telotrophic ovarioles in the ovary varied greatly among different insect species belonging to this family. For example, the amount of ovarioles in a ovary was 6 in *Cicadella viridis*, 5 in *Kolla paulula*, 12 in *Exitianus indicus*, 6 in *Psammotetttix striatus*, 8 in *Japananus hyalinus*, 8 to 10 in *H*. *coagulata* [13, 29–30]. In the present study, we found 4 ovarioles in *M*. *onukii* female adult ovary (Fig 2). Generally, according to the previous reports, the middle or the end part of oviduct usually swelled to form the ball-shaped spermatheca in Cicadelliadae [13, 29–30]. Differently, the nearly heart-shaped spermatheca of *M*. *onukii* female adult was identified in our study, of which one side was relatively flat and the other side has two protuberant structures (Fig 3A). We noticed that the *M*. *onukii* oviducts, the bottom of which was slightly-expanded, had a small amount of fluid substance, especially when the reproductive system was at stages II, IV (Fig 3A). We guess that the fluid substance might be sperm, which need to be further verified.

As shown in Fig 3a, for the female adult that copulated for continuous 3 day and the ovary maturity was at stage IV, we found a mature oocyte was in the middle of the oviduct. Combined with the results of the egg-laying data, we found that it took at least 3–4 d for the oocyte to mature and form the zygote (Fig 3A and 3C). Morphological results of the ovary at different stages showed that once the mature oocyte appeared, protuberant structures on the spermatheca were disappeared in turn, in most cases (Fig 3A). Similar result has been reported previously [28]. Generally, there are two patterns of oocyte development: one is synchronous, the other is asynchronous, which are different with the herbivorous pest species; moreover, the oocyte development pattern was found to be related with the egg-laying mode [13–15, 31–33]. For example, the development of oocytes in the ovary of the queensland fruit fly (*Strumeta tryoni*) and glassy-winged sharpshooter (*H*. *coagulata*) are synchronous, thus the egg laying mode of them are mass spawning [13–15, 31]. While the development of oocytes in the ovary of Caribbean fruit fly (*Anastrepha suspensa*) and melon fly (*Bactrocera cucurbitae*) are asynchronous, of which oocytes in the ovary matured gradually and laid successively [32, 33]. Similar to this, we found that the oocytes in the ovary of *M*. *onukii* females matured gradually and were laid out singly in different batches (Fig 3; S1 Fig). The asynchronous development of oocytes among different ovarioles became conspicuous when the ovaries were at stages III, IV (Fig 3A). For example, the maturity of oocytes were in different levels when the ovary at stage III, of which one of them started to have yolk deposition ahead of others; both a few immature oocytes in the different maturity levels and mature oocytes in the ovarian calyxs, lateral oviduct, common oviduct, or ovarioles were observed when the ovary at stage IV. Therefore, the development of oocytes in the ovary of *M*. *onukii* is asynchronous. Based on the results of the rhythm investigation, we found that a single *M*. *onukii* female adult laid eggs up to 2–3 per day (Fig 5). The above results successfully demonstrated the oviposition characteristics of the *M*. *onukii* female adult, and explain the reasons why *M*. *onukii* is difficult to control from the insect morphological and physiological point of views.

Similar to our results, Yao et al. (2020) found that the *M*. *onukii* female adults deposited eggs throughout the day, and the egg amount laid by female adults started to increase from 12:00 at noon, then greatly decreased at 24:00 in a day [23]. A number of studies showed that the egg-laying behavior of *M*. *onukii* were influenced by tea varieties, temperature, light, intercropping and shading in tea plantation [24, 25, 34–36]. Therefore, the experimental designs adopted by different studies are supposed to influence the results to some extent. In the previous study, 60 tea green leafhoppers that were randomly selected regardless of gender and three tea shoots were set for the investigation of oviposition rhythm [23]. While, in our study, 4 *M*. *onukii* female adults with fully 8 d copulation experience together with one tender tea shoot were used for the rhythm survey. Interestingly, the results of both studies showed that the egg amount laid by female adults from 12:00 to 24:00 was clearly greater than that from 00:00–12:00. Taken together, we assumed that *M*. *onukii* female adults may indeed prefer to lay eggs during 12:00 to 24:00.

The abdomen of *M*. *onukii* female adult at stage III or IV was found to emit green fluorescence using BLDM [23, 27]. We guessed there was positive correlation between the fluorescence strength and the number of mature and immature oocytes in the ovary. To support this assumption, an ingenious verification was conducted. Firstly, the inner reproductive system of tea leafhopper female adult was carefully dissected under the stereomicroscope. Secondly, all light sources of the stereomicroscope were turned off and the eyepieces were blocked with a blue filter. Thirdly, blue light beam was used to illuminate the inner reproductive system directly. Finally only mature or immature oocyctes in the inner reproductive system were found to emit green fluorescence, and the rest parts of the inner reproductive system were dark red (Fig 2B and 2C), which suggested that the fluorescent substance of *M*. *onukii* egg probably derived from the yolk. Our results provide solid proof for the new egg detection method—BLDM, from the point of insect physiology.

## Conclusion

In the current study, we clarified the main structures of the inner reproductive system of tea green leafhopper female adult. Oviposition behavior was clearly divided into six oviposition steps. According to the maturity of oocytes, the maturity stages of the reproductive system under different copulation periods were classified into 4 stages. For female adults with the high maturity reproductive system (stage IV), mature and immature oocytes were presented simultaneously, and the developmental levels of oocytes were asynchronous among different ovarioles. The proportion of gravid females with oocytes significantly increased with the continuous copulation time. In sync with the development of the inner reproductive system, female adults slightly deposited eggs at the 5th day, and then the egg number increased significantly. In addition, we found, whether mature or immature, oocytes in the ovarioles always emitted green fluorescence under blue light excitation, which in turn provide solid proof for the new egg detection method from the insect physiology point of view. Our study provided intuitive evidences for proving the biological characteristics of oogenesis and egg-laying of tea green leafhopper, which will be helpful for developing new genetic control methods.

## Supporting information

S1 Fig *Matsumurasca onukii* and their damage in tea plantation. (1) Adult feeding; (2) Egg-laying; (3) Eggs; (4–5) Damage.
(TIF)

S2 Fig Feeding and mating devices for *Matsumurasca onukii*. (A) Rearing cages; (B). Rearing tubes for individual *M*. *onukii* nymphs; (C) Glass tubes for conducting mating treatment.
(TIF)

S1 Video Oviposition behavior of the *Matsumurasca onukii*. (MP4)
