# Supplementary figures and images for: Biological evidences for successive oogenesis and egg-laying of Matsumurasca onukii
Source: PLoS One. 2022 Feb 17;17(2):e0263933. doi: 10.1371/journal.pone.0263933 (PMC8853495; doi:10.1371/journal.pone.0263933)

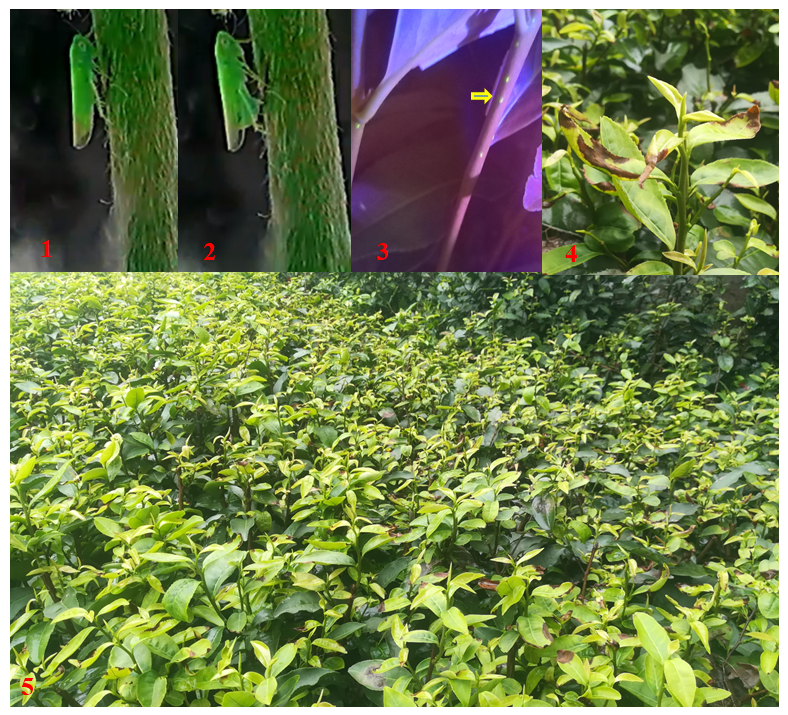

Supplement: S1 Fig — (1) Adult feeding; (2) Egg-laying; (3) Eggs; (4–5) Damage. (TIF) [file pone.0263933.s001.tif]

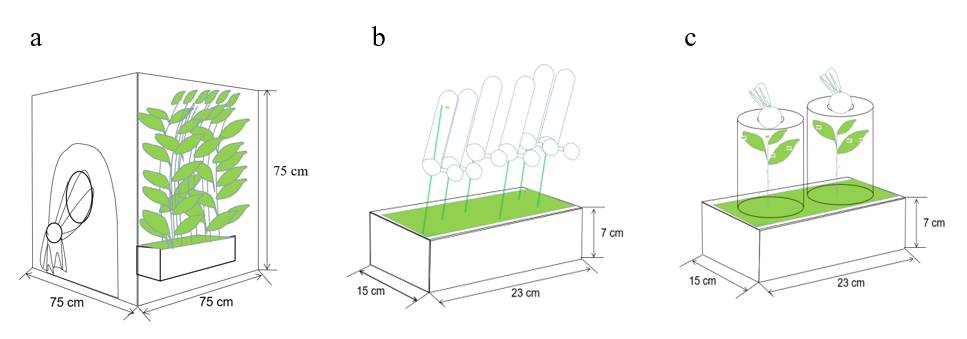

Supplement: S2 Fig — (A) Rearing cages; (B). Rearing tubes for individual M. onukii nymphs; (C) Glass tubes for conducting mating treatment. (TIF) [file pone.0263933.s002.tif]
